# Axillary right vein percutaneous ballooning in hemodialysis catheter replacement: A case report

**DOI:** 10.1177/11297298261476292

**Published:** 2026-09-18

**Authors:** Ferrara Gaetano, Aucella Francesco, Nasuto Michelangelo, Gatta Giuseppe, Ciccarese Giovanni, Aucella Filippo

**Affiliations:** 1 Department of Nephrology and Dialysis, Scientific Institute for Research and Health Care, Fondazione Casa Sollievo della Sofferenza, San Giovanni Rotondo, Italy; 2 Radiology Interventional Unit, Scientific Institute for Research and Health Care, Fondazione Casa Sollievo della Sofferenza, San Giovanni Rotondo, Italy

**Keywords:** Axillary vein, PTA ballooning, catheters, dialysis access, ultrasonography, Doppler evaluation, vascular access rescue, interventional radiology

## Abstract

Reliable vascular access remains a key point of hemodialysis therapy although each patient’s journey through chronic kidney disease is unique. The 2019 Kidney Disease Outcomes Quality Initiative (KDOQI) vascular access guidelines introduced a patient-centered approach, emphasizing individualized vascular access planning based on each patient’s clinical characteristics, comorbidities, life expectancy, and treatment goals. Vascular access troubleshooting is required when the creation of a native arteriovenous fistula or prosthetic graft is not feasible, necessitating the use of tunneled central venous catheters inserted into central veins such as the internal jugular, subclavian, axillary, or femoral ones. The internal jugular vein is generally considered the preferred access site. However, when its use is precluded by thrombosis, stenosis, or unfavorable cervical anatomy, the axillary vein represents a valuable alternative. In this report, we describe the replacement of a tunneled hemodialysis catheter in the right axillary vein following percutaneous balloon angioplasty of an early axillary vein stenosis that had developed 6 months after the first catheter placement. This strategy was adopted because all other upper central venous access sites were unavailable owing to right internal jugular vein stenosis and previous thrombosis of the brachiocephalic vein. Post-procedural chest radiography confirmed the correct positioning of the catheter tip within the right atrium site. The patient successfully resumed maintenance hemodialysis without further catheter dysfunction during follow-up. This case highlights the feasibility of percutaneous balloon angioplasty of a previously cannulated axillary vein to preserve vascular access and facilitate catheter replacement in hemodialysis patients with multiple comorbidities and severely limited central venous access options.

## Introduction

A challenging vascular access scenario arises when the creation of a native arteriovenous fistula (AVF) or the implantation of a prosthetic arteriovenous graft for hemodialysis is not feasible because of reduced vessel caliber, severe vascular calcification, central or peripheral venous stenosis, thrombosis or failure of previous vascular accesses.^1^

In these circumstances, a tunneled central venous catheter made of polyurethane or silicone may be inserted into the central venous system to provide long-term vascular access for hemodialysis.

In selected complex cases, the axillary vein may represent a valuable alternative access site. The axillary vein is the continuation of the basilic vein and is anatomically divided into three segments by the pectoralis minor muscle: proximal, posterior, and distal.^2^

Several studies have demonstrated that axillary vein cannulation can be safely performed using anatomical landmarks, palpation, or, preferably, real-time ultrasound guidance.

Nevertheless, axillary vein stenosis may develop as a consequence of repeated catheterization, prolonged catheter dwell time, vascular trauma, chronic inflammation, or thrombosis. This complication may result in catheter dysfunction and inadequate dialysis delivery. In addition, thrombus formation or the development of a fibrin sheath surrounding the catheter may progressively organize into fibrous tissue, leading to partial or complete venous obstruction. Malposition of the catheter tip and the presence of an ipsilateral arteriovenous fistula may further increase the risk of central venous stenosis. Progressive venous outflow obstruction can promote the formation of collateral venous circulation and may manifest clinically as ipsilateral upper-limb edema, pain, and venous hypertension.^3^

Percutaneous transluminal angioplasty (PTA) represents the first-line endovascular treatment for symptomatic axillary vein stenosis identified by imaging, with the aim of restoring venous patency, preserving vascular access, and maintaining adequate hemodialysis.

## Case presentation

We report the case of a 64-year-old man receiving maintenance hemodialysis who was admitted to our Nephrology Unit because of malfunction of a tunneled right axillary venous catheter that had been implanted 6 months earlier. The catheter had been placed through the right axillary vein because conventional upper central venous access sites were no longer available and the patient was not considered a suitable candidate for either a native arteriovenous fistula (AVF) or a prosthetic arteriovenous graft. Duplex ultrasound demonstrated a mid-forearm arterial and venous diameter of approximately 2 mm, with extensive arterial calcification.

The patient’s medical history was significant for diabetes mellitus, chronic asthmatic bronchitis, and previous vascular access failure, including thrombosis of a synthetic expanded polytetrafluoroethylene (ePTFE) graft that had been surgically interposed between the arterialized cephalic vein and the basilic vein following failure of a native AVF.

Regarding vascular access history, the patient initially underwent creation of a native mid-arm AVF in the right upper limb. The fistula matured appropriately and initially provided adequate blood flow.

Three months later, however, the patient developed a critical stenosis of the arterialized cephalic outflow vein. Digital venography confirmed a severe stenotic lesion that was not amenable to percutaneous transluminal angioplasty (PTA). Consequently, an ePTFE interposition graft was surgically implanted between the arterialized cephalic vein and the basilic vein of the right arm.

During this procedure, thrombosis of the right internal jugular vein, previously used for tunneled central venous catheter (CVC) placement, was identified. The tunneled catheter was therefore removed, and a temporary left internal jugular CVC was inserted.

Six months later, the patient was readmitted because of acute thrombosis of the right ePTFE arteriovenous graft. The extent of thrombosis precluded both surgical thrombectomy and endovascular recanalization. Duplex ultrasound demonstrated thrombotic stenosis involving both internal jugular veins and the right femoral vein. In addition, severe bilateral calcification of the radial and ulnar arteries contraindicated the creation of a new prosthetic vascular access because of the high risk of dialysis access-associated steal syndrome (DASS). Ultrasound examination of both upper limbs revealed markedly reduced venous caliber, while the arteries were diffusely calcified. These findings were further supported by thermographic assessment performed during hospitalization.

The patient also had persistent systemic hypotension trend, both during hemodialysis sessions and on outpatient blood pressure measurements.

Pre-procedural venography demonstrated patency of the right brachial, axillary, subclavian, and brachiocephalic veins. Nevertheless, the patient categorically refused the creation of another prosthetic graft in either the ipsilateral or contralateral upper limb because of the early failure of the previous ePTFE graft.

Based on these findings, a tunneled polyurethane hemodialysis catheter (15.5 F; length 19–23 cm, Joline GmbH & Co. KG) was successfully inserted through the right axillary vein under real-time ultrasound guidance using the Seldinger’s technique. During the procedure, the patient was positioned supine with the right arm abducted on 90° and externally rotated to maximize the transverse diameter of the axillary vein.

The catheter provided adequate blood flow throughout subsequent hemodialysis sessions, allowing removal of the temporary femoral catheter.

Ten months later, the patient was readmitted because of recurrent catheter dysfunction characterized by repeated blood pump alarms occurring approximately 2 h after hemodialysis session start.

Laboratory tests at admission showed normal hemoglobin concentration, leukocyte count, platelet count, serum potassium, calcium, iron profile, lipid profile, and international normalized ratio (INR).

Blood urea nitrogen (BUN), serum creatinine, phosphorus, and blood glucose levels were elevated.

A nasopharyngeal swab for SARS–CoV-2 was negative.

An initial attempt to exchange the tunneled right axillary catheter over a guidewire was unsuccessful. Consequently, a temporary right femoral venous catheter was inserted, while the malfunctioning axillary catheter was left in place.

Antegrade venography through the right axillary catheter demonstrated extensive collateral venous circulation within the right upper limb, occlusion of the basilic vein, and a significant stenosis of the mid-axillary vein located approximately 5 cm from the skin entry site. Contrast medium reached the superior vena cava through collateral venous drainage involving the thyroid venous plexus (Figures 1(a) and (b) and 2(a) and (b)).

**Figure 1.**
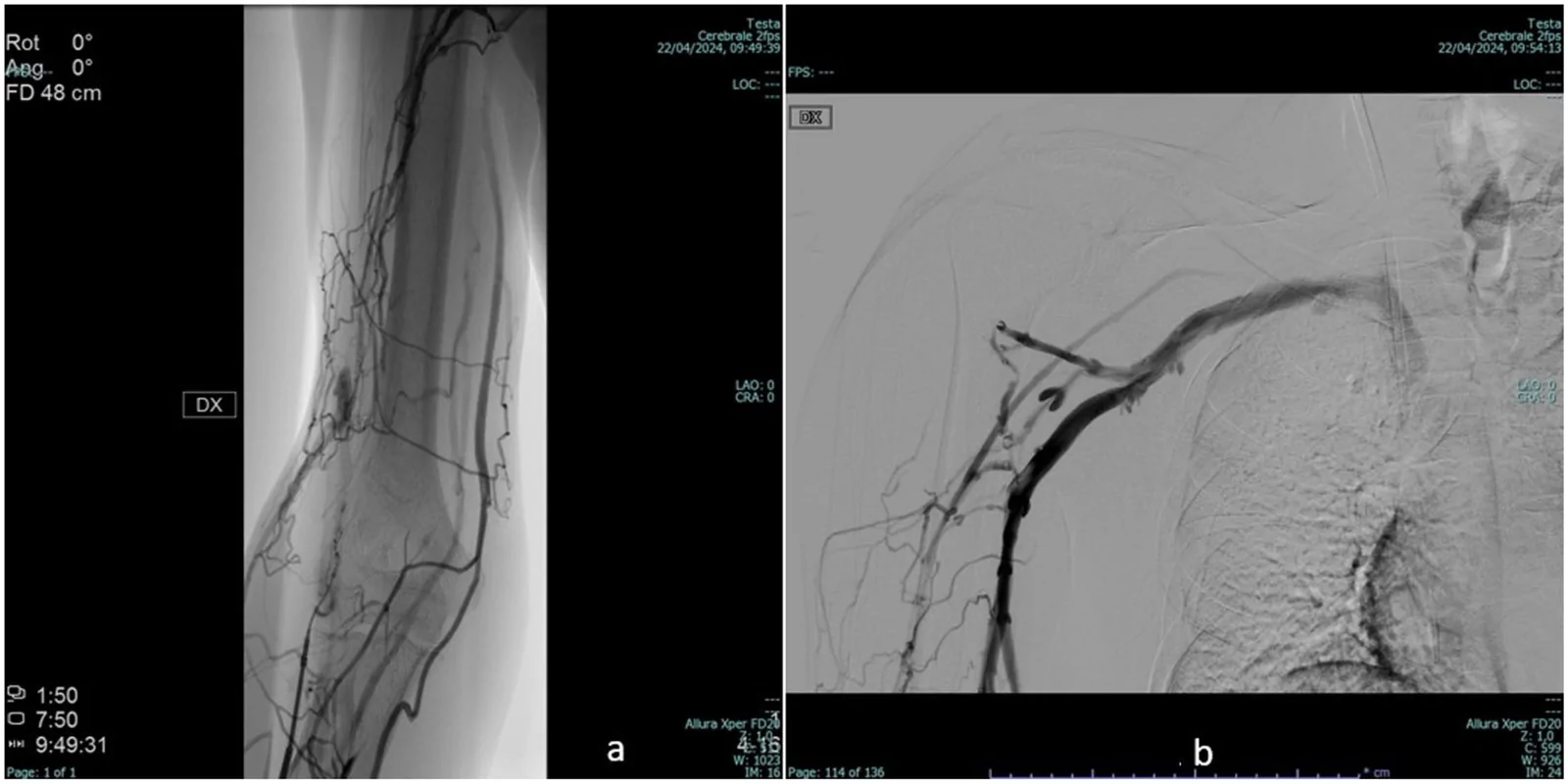
Diagnostic flebography showing occlusion of right cefalic vein (a) and stenosis of proximal axillary vein (b).

**Figure 2.**
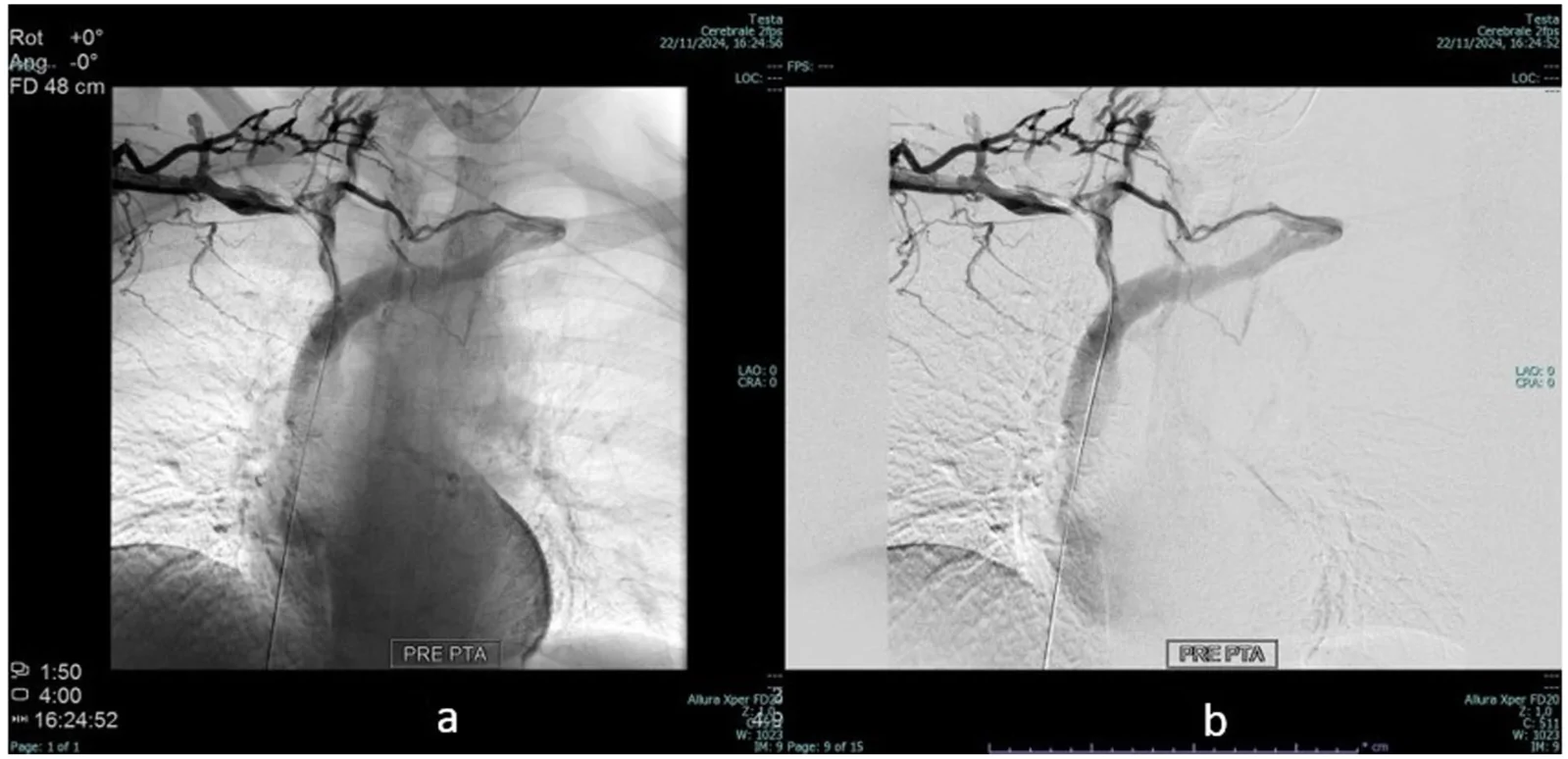
Diagnostic flebography of right arm in 90° abduction position. Infraclavicular subclavian vein access (a) and critical stenosis of right axillary vein with associated opacification of various collateral veins (b).

The patient subsequently underwent catheter replacement in the Interventional Radiology Unit. The procedure included percutaneous transluminal balloon angioplasty of axillary vein stenosis (Armada™ 35 balloon catheter, 6.0 × 100 mm × 80 cm, 5 F; Abbott Vascular) followed by mechanical fibrin sheath disruption (inflation pressure at 16 atm using an Encore™ 26 inflation device; Boston Scientific; Figure 3(a)). The procedure resulted in immediate restoration of venous patency, with prompt resolution of the stenosis and unobstructed contrast flow during the venous phase (Figure 3(b)).

**Figure 3.**
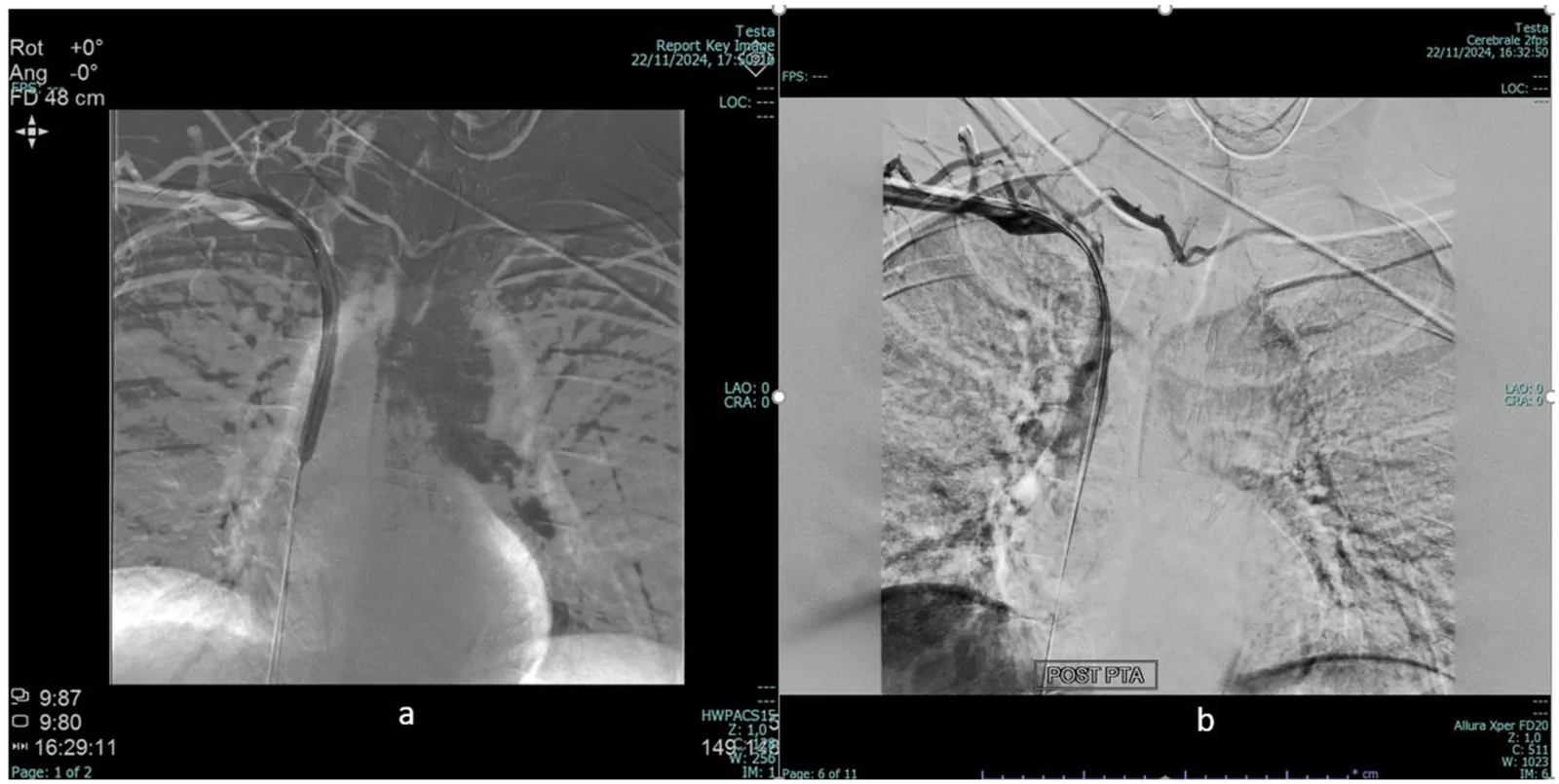
Axillary right vein balloon PTA (6 × 100 mm (a)) and post-PTA flebography (b).

A new tunneled polyurethane hemodialysis catheter (Joline GmbH & Co. KG; 15.5 F; length 19–23 cm, tunneled) was then successfully implanted through the right axillary vein using the Seldinger technique under fluoroscopic guidance after PTA flebography.

Post-procedural fluoroscopy confirmed correct positioning of the catheter tip within the right atrium. Catheter blood flow was immediately satisfactory, allowing removal of the temporary femoral catheter.

Eleven months later, the patient was admitted because of right axillary tunneled catheter exit-site suspected infection. Clinical examination revealed no fever during or between dialysis sessions. Inflammatory markers were within the normal range, and microbiological culture of the exit-site swab was negative. The patient was successfully managed conservatively with local wound care using a sodium hypochlorite solution. Neither systemic antibiotic therapy nor catheter removal was required.

At the most recent follow-up, the patient undergoes regular maintenance hemodialysis using the same tunneled right axillary catheter, which remains fully functional.

## Discussion

Approximately 50,000 patients in Italy currently undergo maintenance hemodialysis. Native arteriovenous fistula (AVF) remains the preferred vascular access and is considered the gold standard because of its superior long-term patency and lower complication rates.

Current vascular access strategies have shifted toward a personalized, patient-centered approach, as recommended by the latest K-DOQI guidelines. Although AVFs and prosthetic arteriovenous grafts remain the preferred options, their long-term success depends on appropriate patient selection, surgical expertise, regular surveillance, timely maintenance, and early referral for vascular access creation.

When conventional vascular access is no longer feasible because of multiple comorbidities or exhausted vascular access sites (including central venous thrombosis or stenosis, recurrent infections, tracheostomy, diffuse arterial calcification, or inadequate venous caliber resulting from previous cannulation or repeated venipuncture) tunneled central venous catheters may represent the only available option. In these circumstances, alternative access sites such as the axillary vein should be considered (Table 1).

**Table 1.** Major vascular access Comparison.

Access	Advantages	Disadvantages
Internal jugular	Low infection risk	Limited mobility, thrombosis
Femoral	Easy access	Infections, immobilization
Subclavian	Comfort	Pneumothorax, pinch-off syndrome
Axillary	Extrathoracic safety, comfort, alternative access	More technically complex procedure

Ultrasound-guided infraclavicular axillary vein cannulation offers several advantages over more conventional central venous access sites. These include a lower risk of pneumothorax, avoidance of pinch-off syndrome, continuous real-time ultrasound visualization during puncture, a stable catheter exit site, fewer mechanical complications, a shorter subcutaneous tunnel, improved patient comfort, and potentially lower rates of catheter-related bloodstream infection.^4^

Conversely, the subclavian vein is generally avoided in patients requiring long-term hemodialysis because of the substantial risk of central venous stenosis, which may permanently compromise future vascular access options.

In this case, the right axillary vein was therefore selected after careful evaluation of the patient’s vascular access history and the unavailability of conventional central venous access sites.

Several authors have described more unconventional vascular access routes for patients with exhausted vascular access, including translumbar, transhepatic, and azygos vein catheterization. However, these highly specialized techniques were not indicated in our patient because the axillary vein remained a feasible access route.

Among recently developed endovascular techniques, the Surfacer^®^ Inside-Out^®^ Access Catheter System has emerged as a promising option for patients with chronic thoracic central venous occlusion (CTVO), a condition frequently associated with repeated or prolonged central venous catheterization. The procedure is usually performed through the right femoral vein, although successful transcollateral approaches have also been reported in patients with completely exhausted vascular access. This technique creates a new central venous pathway when conventional routes are no longer available.^5^

Successful axillary vein cannulation requires specific operator expertise and should ideally be performed under real-time ultrasound guidance. Once vascular access has been established, subsequent catheter exchange may be facilitated by endovascular techniques, including fibrin sheath disruption and balloon angioplasty.

The axillary vein can be visualized in both short- and long-axis ultrasound views. Previous studies have shown that arm abduction and external rotation increase the transverse diameter of the vein, thereby facilitating cannulation.^6^ Likewise, an appropriately directed infraclavicular subcutaneous tunnel toward the deltopectoral groove may optimize catheter positioning and reduce the risk of kinking.

The infraclavicular location, favorable vessel diameter, and excellent ultrasound visualization make the axillary vein an attractive alternative for tunneled hemodialysis catheter placement. Although anatomical landmark and palpation techniques have been described, ultrasound guidance is currently considered the standard of care because it improves procedural success and reduces complications.^7^

Many reports have demonstrated high technical success rates, satisfactory dialysis blood flow, and good long-term catheter performance following axillary vein cannulation, with a relatively low incidence of central venous stenosis. When early stenosis develops, percutaneous transluminal angioplasty under fluoroscopic guidance represents an effective strategy for restoring catheter function while preserving vascular access.

Our case further supports the feasibility of balloon angioplasty for early axillary vein stenosis associated with tunneled hemodialysis catheters. This approach allowed successful catheter replacement while preserving a valuable vascular access site in a patient with severely limited central venous access options.

In addition, maintaining axillary vein patency may help prevent progressive central venous hypertension and the development of superior vena cava syndrome (SVCS) in hemodialyzed patients requiring long-term central venous catheterization.

The management of SVCS in HD patients can be challenging and a decision on how to achieve a safe and functional VA should be tailored to the patient’s conditions and individual status such as therapeutic resources. Although several therapeutic options are available and the basic principles are well established in the literature (several treatment options were identified and assessed using current international VA guidelines and recently published studies showed level of evidence C, not higher) it has been reported a stepwise approach to SVCS. First of all an upper extremity AVF should be preserved or made functional. Endovascular treatment of central vein stenosis or occlusion (CVSO) should primarily consist of balloon dilatation. Placement of a stent or stent graft should be considered as a secondary option.

Hemodialysis Reliable Outflow (HeRO^®^ grafts) and the Surfacer^®^ Inside-Out^®^ Access Catheter System are specialized medical systems that could be considered facing recurrent CVSO and recanalization with a Surfacer may provide further options for tCVC or even HeRO application. Lower limb native fistula or graft may be considered as an alternative for certain HD patients when the risk of lower limb ischemia and infection has been deemed.^8^

Although the long-term durability of this strategy remains to be established, our experience confirms the feasibility and safety of axillary vein percutaneous transluminal angioplasty (PTA) followed by tunneled catheter replacement. This approach represents an effective vascular access salvage technique for late-referral maintenance hemodialysis patients with exhausted access options.

## Data Availability

In his own medical record.
